# Continuous VOCs Monitoring in Saturated and Unsaturated Zones Using Thermal Desorber and Gas Chromatography: System Development and Field Application

**DOI:** 10.3390/ijerph19063400

**Published:** 2022-03-14

**Authors:** Jinsung An, Dong-Jun Baek, Jiseok Hong, Eunsoo Choi, Ijung Kim

**Affiliations:** 1 Department of Biological and Environmental Engineering, Semyung University, Jecheon-si 27136, Korea; jsan@semyung.ac.kr (J.A.); hwwdong123@naver.com (D.-J.B.); 2 Department of Civil and Environmental Engineering, Hongik University, Seoul 04066, Korea; ghddptjr@gmail.com (J.H.); eunsoochoi@hongik.ac.kr (E.C.)

**Keywords:** BTEX, on-site monitoring, saturated zone, unsaturated zone, oil-contaminated site

## Abstract

Subsurface VOC monitoring has been mainly based on manual sampling, transport, and analysis, which would require a sufficient amount of samples to ensure data accuracy and reliability, and additional costs to ensure sample quality. Therefore, a continuous on-site monitoring system is desirable for accurate measurement and subsequent risk assessment. In this study, benzene, toluene, ethylbenzene, and xylene (BTEX) were continuously monitored by the system based on a thermal desorber (TD) and gas chromatography (GC) in an oil-contaminated site that consisted of saturated and unsaturated zones. For the saturated zone, fully automated groundwater sampling and purging processes were performed, and the gasified samples were applied to the TD–GC system. For the unsaturated zone, the gaseous sample in the site was directly applied to the TD–GC system. After verifying the accuracy and precision of the monitoring system, the continuous monitoring system was successfully operated for more than a month in the field. The monitoring system used in this study is applicable to other sites for continuous monitoring, thus providing a scientific background for advanced risk assessment and policy development.

## 1. Introduction

Volatile organic compounds (VOCs) generally enter the soil and groundwater from external sources [1,2]. Most oil-contaminated sites are found in or near military bases, gas stations, and railroad depots [3,4,5]. Once introduced into the soil, gaseous VOCs are generated continuously and can enter humans via vapor intrusion, consequently leading to chronic exposure through VOC inhalation [6]. Since the adverse effects of VOCs on health were officially announced (e.g., classification of benzene as carcinogenic to humans (Group 1) by the International Agency for Research on Cancer Monographs [7]), more attempts have been made to monitor VOCs underground [8,9,10].

VOC monitoring has mainly been conducted during on-site sampling, which is followed by laboratory analysis [11,12]. Such monitoring inevitably requires transfer time of the samples along with pretreatment (sometimes conducted), possibly altering the original sample quality. Thus, the demand for fast and continuous VOC monitoring is increasing; consequently, more attention has been paid to portable VOC sensors. As a representative portable VOC sensor, the photoionization detector (PID) has become commercially available; additionally, the combination of PID with gas chromatography (GC) has been recently investigated for monitoring VOCs in the water and air [13,14]. However, most PID sensors are non-selective to a particular VOC, and the detection limit is relatively lower than that of conventional detectors, such as flame ionization detectors (FIDs) [15]. Since the regulations on VOCs in natural and built environments are often established by individual components such as benzene and toluene [16,17], separate detection of the representative VOC components (e.g., BTEX) with high accuracy is required for continuous VOC monitoring. In fact, the individual VOC components have been determined by a thermal desorber (TD) and GC system in indoor air quality monitoring [18,19], and it is desirable to apply such a system in the soil and groundwater. Since VOC monitoring in the subsurface has been conducted either in the soil or in groundwater [20,21], it is necessary to monitor VOCs simultaneously in both the soil and groundwater to determine VOC migration and mitigation in the subsurface.

In this study, a continuous benzene, toluene, ethylbenzene, and xylene (BTEX) monitoring system for both saturated and unsaturated zones was developed using a combination of TD and GC–FID for field applications. Specifically, the accuracy and reliability of the system were thoroughly examined to analyze the BTEX components separately. The system was finally applied as a continuous on-site monitoring system to a site contaminated with oil spills. Therefore, the feasibility of the on-site BTEX monitoring system was examined for both saturated and unsaturated zones at the field.

## 2. Materials and Methods

### 2.1. System Configuration

**Figure 1:**
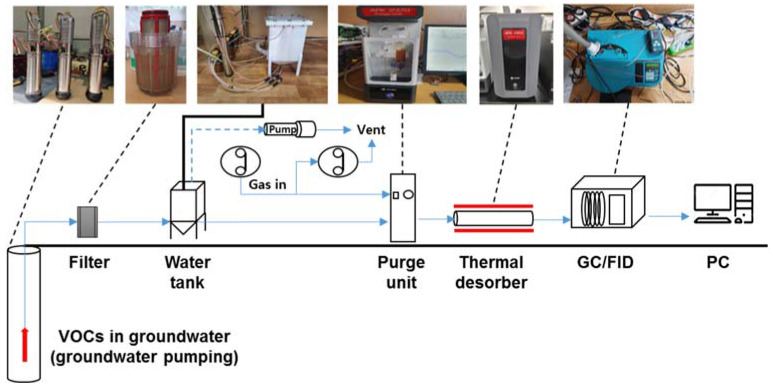
System configuration to monitor BTEX concentrations in the saturated zone.

The overall schematics of the BTEX monitoring system, including purge units (APK2370, KNR, Inc., Namyangju, Korea), TD (APK2350, KNR, Inc., Namyangju, Korea), and GC–FID (200 Series GC, Ellutia, Witchford, UK), in the saturated zone is shown in Figure 1. A submersible pump (HSP1-4, Haedong pump, Busan, Korea) was used to pump the groundwater sample from a well. Subsequently, the sample was collected in a water tank after passing it through a stainless-steel mesh filter (pore size = 80 mesh). After pumping the groundwater for 8 min (approximately 10 L of sample collected), the sample was introduced into a purge unit at a flow rate of 38 mL/min for 0.6 min. Subsequently, the purge gas (N_2_) was injected at a flow rate of 80 mL/min for 12 min. The analysis conditions for TD and GC/FID were as follows: The TD was equipped with a focusing trap filled with Tenax-TA, which is specialized for use during the adsorption and desorption of gaseous VOCs. The focusing trap was frozen to −20 °C for VOC adsorption and heated to 320 °C for VOC desorption. Moreover, the valve oven and transfer line of the TD were maintained at 150 °C and 180 °C, respectively. The GC was equipped with an FID and a chromatography column (VB624, Vici Valco). The column flow was maintained at 1 mL/min, and the column temperature was held at 40 °C for the first 5 min; subsequently, the temperature was heated to 100 °C at a rate of 10 °C /min and then to 250 °C at a rate of 20 °C /min. The GC inlet and detector temperatures were maintained at 250 °C and 300 °C, respectively, during the analysis period. N_2_ gas (99.99%, Seoul Specialty Gas Co., Ltd., Seoul, Korea) was used as the purging and carrier gas. This series of processes was developed to be fully automatic.

**Figure 2:**
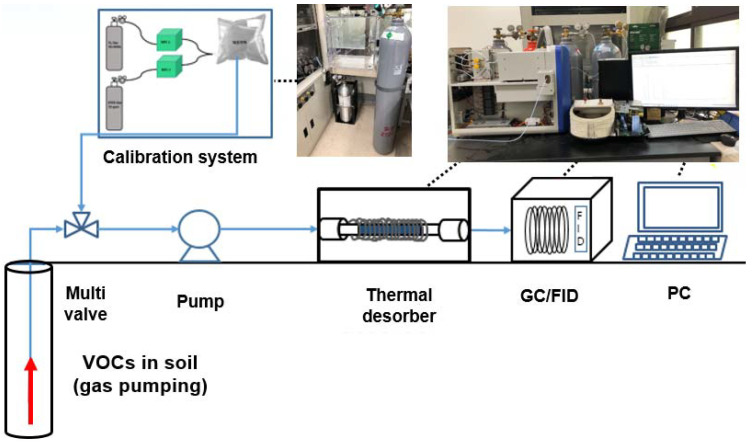
System configuration to monitor gaseous BTEX concentration in the unsaturated zone.

Figure 2 shows an overall schematic of the monitoring system. TD and GC were combined for BTEX determination in the unsaturated zone. Prior to the TD, a multi-valve and pump were used along with a mass flow controller to provide the sampling gas at constant flow rates of 50, 100, and 150 mL/min according to the test conditions. For the field test, the flowrate and the adsorption time were maintained at 50 mL/min and 5 min, respectively. The analysis conditions for TD and GC/FID were the same as those used for the saturated zone, except the chromatography column (DB-5, Agilent).

### 2.2. System Calibration and BTEX Measurement

The system was calibrated manually for the BTEX analysis in the saturated zone. The multi-VOC standard (100 μg/mL) (Accustandard, S-7686-R4-0.1X, New Haven, CT, USA) was diluted to 10, 40, and 80 μg/L using distilled water (DI) to prepare working standards. The purge unit connected to the water tank was pulled out, washed with DI three times, and immersed in the prepared working standards. The accuracy of the calibration curve was validated by measuring 40 μg/L of BTEX solution every week in the field. Subsequently, it was confirmed that the retention time of the target analyte in the chromatogram was delayed.

The calibration system for the unsaturated zone was prepared separately and connected to the monitoring system via a multi-valve, which selected the calibration gas flow as scheduled in the program. Standard 100 ppb BTEX gas, which was used as the calibration gas, was prepared by mixing 10 ppm BTEX gas (RIGAS Co., Ltd., Daejeon, Korea) and 99.99% N_2_ gas via two mass flow controllers (Wiz-701, KBM Tech Co., Ltd., Incheon, Korea) at a rate of 10 mL/min and 990 mL/min, respectively. The BTEX gas was stored in a 30-L Tedlar bag (Top Trading Eng Co., Ltd., Seoul, Korea), and then supplied to the system directly by connecting the bag to the multiple valves of the system inlet line. To minimize the exposure of the Tedlar bag to the ambient air, the entry/exit valve of the Tedlar bag was immediately opened and closed after the injection of the standard BTEX gas was initiated and completed. Instead of varying the BTEX gas concentration, the amount of adsorbed BTEX gas was gradually increased by varying the adsorption time to 5, 10, 15, 20, and 25 min. The five points were linearly regressed to provide a calibration curve with a slope. Additionally, a conversion factor between the adsorption amount and the raw data (current measured in pA) were obtained from the GC analysis. When the coefficient of the determinant (R^2^) was less than 0.98, the system and the measurement method were thoroughly checked, and the calibration was repeated until the R^2^ value was greater than 0.98. During the continuous field test, the Tedlar bag was replaced every two weeks to minimize variations in the BTEX concentration in the Tedlar bag. The conversion between the BTEX concentration and the amount of adsorbed BTEX was calculated using Equation (1):

(1) $$m_{BTEX}=\frac{C_{BTEX}\times M_{W}\times Q\times t}{V_{M}}$$

where $m_{BTEX}$ is the adsorbed mass of BTEX (ng), $C_{BTEX}$ is the BTEX concentration (ppb), $M_{W}$ is the molecular weight of BTEX (g/mol), Q is the flow rate (L/min), t is the adsorption time (min), and $V_{M}$ is the theoretical molar volume (L/mol). The calibration curve of the four separate components (benzene, toluene, ethylbenzene, and *m,p*-xylene) of the BTEX gas was obtained using the adsorbed mass and the corresponding GC peak area.

### 2.3. Verification of the Monitoring System

To ensure the validity and reliability of the monitoring system, the system was examined using standard BTEX gas (100 ppb) under different sampling flow rates and sample adsorption times to the cold trap (an adsorber tube filled with Tenax-TA) in the TD. The sampling flow rate and adsorption time varied from 50 to 150 mL/min and from 5 to 20 min, respectively. At each condition, continuous measurements were taken until the BTEX concentration reached a plateau, after which at least seven measurements were taken to calculate the precision and accuracy, as shown below [22].

(2) $$Precision=\frac{Standard\;deviation\;of\;the\;measurements}{Average\;of\;the\;measurements}\times 100\left(\%\right)$$

(3) $$Accuracy=\frac{Average\;of\;the\;measurements}{True\;value}\times 100\left(\%\right)$$

In the case of the saturated zone monitoring system, working standards (2 and 4 μg/L) were analyzed at least seven times to calculate the precision (i.e., relative standard deviation and the limit of detection, LOD). In addition, the accuracy of the system was calculated by repeated measurements using 10, 20, and 40 μg/L working standards.

### 2.4. Field Study

**Figure 3:**
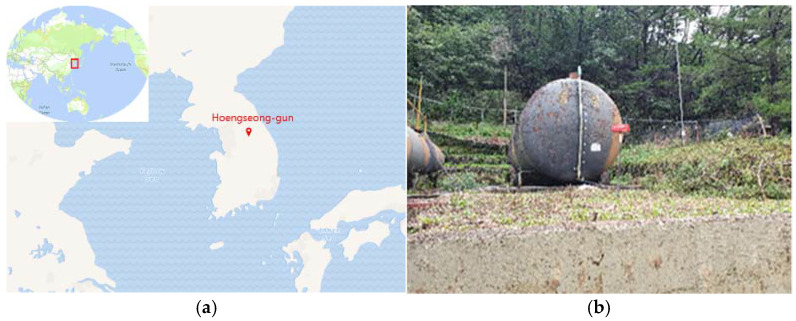
(**a**) Site location (Hoengseong) in Republic of Korea and (**b**) the contamination source (a rusted oil tank) at the site. Additional pictures of the monitoring wells at the site are shown in Figure S1.

After system verification, an identical system was installed in a site that was contaminated with oils. The oil-contaminated site selected for this study was located in a military camp in Hoengseong, Kangwon-do, Republic of Korea (Figure 3). This site has served as a refueling facility for decades; consequently, the prolonged use of the site has led to oil contamination.

BTEX was continuously monitored for more than a month in the site (saturated zone: 24 September 2021 to 10 November 2021; unsaturated zone: 13 October 2021 to 18 November 2021). Table S1 shows the VOC concentrations in the unsaturated zones before the system was installed, indicating the original contamination level of the site.

To assess the relationship between the BTEX concentrations and water characteristics, i.e., turbidity, electrical conductivity (EC), and pH) of groundwater, in situ sensors were installed in the well. In situ sensors for turbidity (Nitro::lyser II), EC (Condu::lyser), and pH (pH::lyser) were obtained from S-CAN (Vienna, Austria). Moreover, precipitation and air temperature data were provided by the Korea Meteorological Administration.

## 3. Results and Discussions

### 3.1. Accuracy and Validation of the System

**Figure 4:**
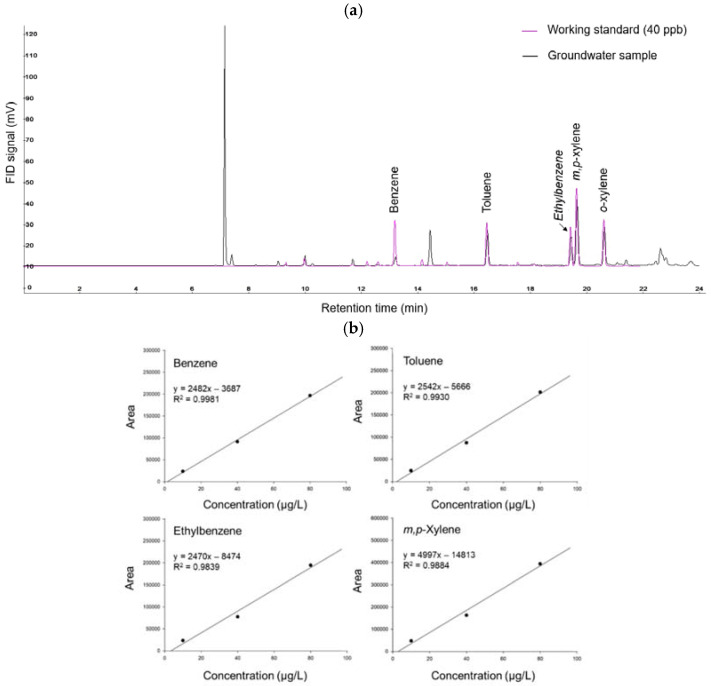
(**a**) Chromatogram of the working standard (40 μg/L) and the groundwater sample and (**b**) calibration curves for BTEX established in the field.

The established purge-TD-GC/FID system successfully separated the BTEX from the groundwater sample (Figure 4a). The calibration curve exhibited good linearity in the range of 10–80 μg/L BTEX concentrations (R^2^ > 0.98) (Figure 4b). The LOD was calculated to assess the detection capacity of the monitoring system. It was defined as three times the standard deviation of the working standards containing BTEX at a low concentration (i.e., 2 μg/L) divided by the slope of the calibration curve. The LOD values for benzene, toluene, ethylbenzene, *m,p*-xylene, and *o*-xylene were 0.056, 0.055, 0.058, 0.075, and 0.073 μg/L, respectively (Table S2). In addition, the accuracy and precision of the system ranged from 98.6% to 101.9% and from 0.97% to 1.41%, respectively (Table S2).

**Figure 5:**
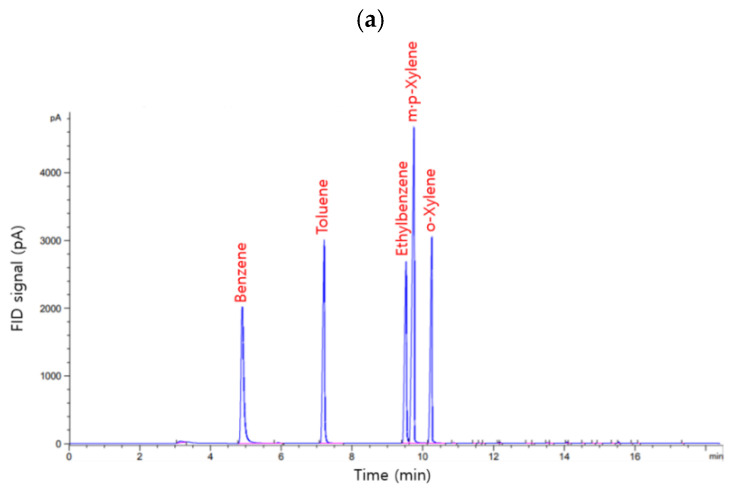

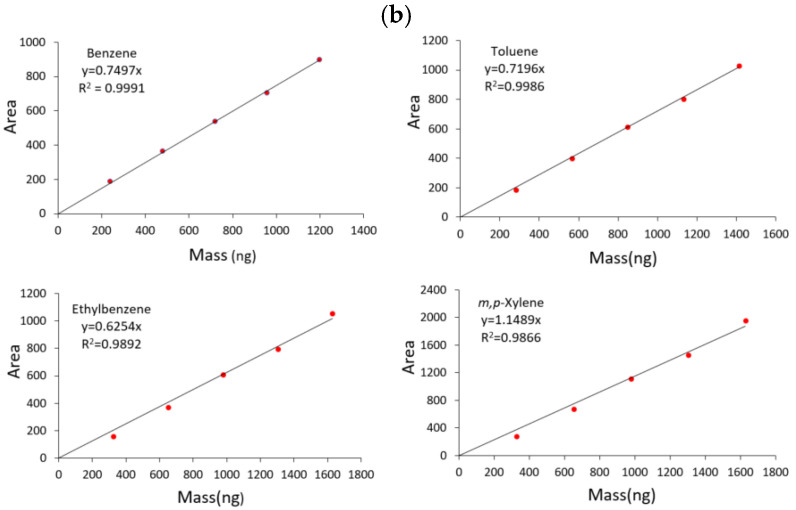
(**a**) Chromatogram of the standard BTEX gas and (**b**) calibration curves for the BTEX gas ($C_{BTEX}$ = 100 ppb, Q = 150 mL/min, *t* = 5, 10, 15, 20, and 25 min).

For the gaseous BTEX analysis, the Tedlar bag filled with 100 ppb BTEX gas was directly connected to the monitoring system. Subsequently, a chromatogram with five clear peaks of benzene, toluene, ethylbenzene, *m,p*-xylene, and *o*-xylene was obtained (Figure 5a). The standard curve showed that the system was reliable (R^2^ > 0.98, Figure 5b), with the accuracy and precision values being within 10% and ±30%, respectively, over the tested conditions (Table S3), thereby meeting the quality control criteria of the standard method for soil contaminant analysis in Korea [22].

### 3.2. Continuous Field Monitoring

#### 3.2.1. Saturated Zone

**Figure 6:**
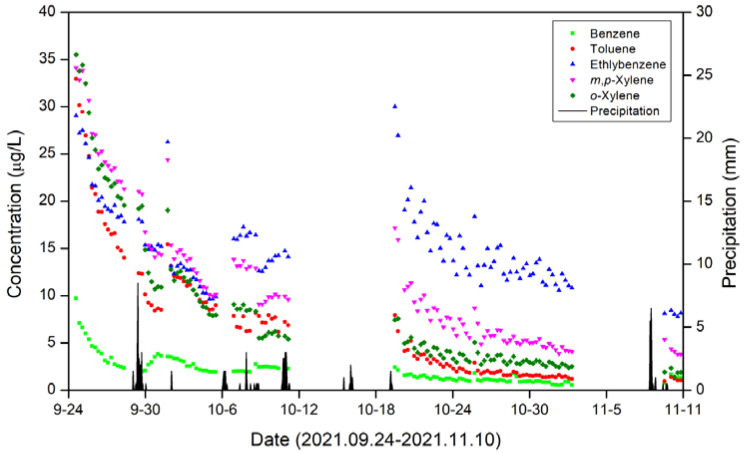
Continuous field monitoring data for BTEX in the saturated zone (monitoring period: 24 September 2021 to 10 November 2021).

The BTEX concentrations obtained from the monitoring system installed in the saturated zone of the study site are shown in Figure 6. Concentrations of benzene, toluene, ethylbenzene, *m,p*-xylene, and *o*-xylene were 0.51–9.71 μg/L, 0.94–32.95 μg/L, 7.76–30.0 μg/L, 3.80–34.11 μg/L, and 1.39–35.50 μg/L, respectively. According to the Korean groundwater quality standards for drinking water (benzene = 0.015 mg/L, toluene = 1 mg/L, ethylbenzene = 0.45 mg/L, and xylene = 0.75 mg/L), the measured concentrations of BTEX during the monitoring period were within the standards. Over the entire monitoring period, a difference of up to 35-fold was observed in the BTEX concentrations. In other words, compared to the existing method, which includes collection, transportation, and laboratory analysis of one sample at a specific time, the concentration determined using an on-site monitoring system enables more reliable risk assessment and/or remediation strategy design.

**Figure 7:**
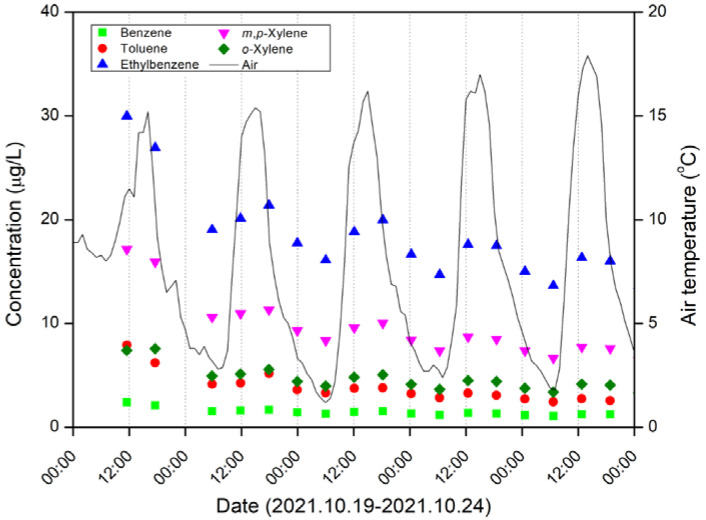
Daily fluctuation patterns of BTEX concentrations, determined using the systems established in this study, and air temperature observations (solid line) conducted during 19–24 October 2021. During this period, no rainfall occurred.

Daily fluctuations of the BTEX concentrations were observed in the groundwater samples (Figure 7). In particular, the concentrations of ethylbenzene and *m,p*-xylene in BTEX noticeably varied according to the changes in the air temperature. The groundwater temperature varied from 14.3 °C to 15.8 °C during the monitoring period (19–24 October 2021), and it was generally maintained at 14.4–14.5 °C. Determining the reason behind the daily fluctuation requires further study. It is expected that a more accurate understanding and insight into the behaviors of BTEX can be obtained through the difference in BTEX concentrations during the day. In other words, the system developed in this study can play an important role in establishing the sampling and remediation strategies of BTEX in oil-contaminated sites.

**Figure 8:**
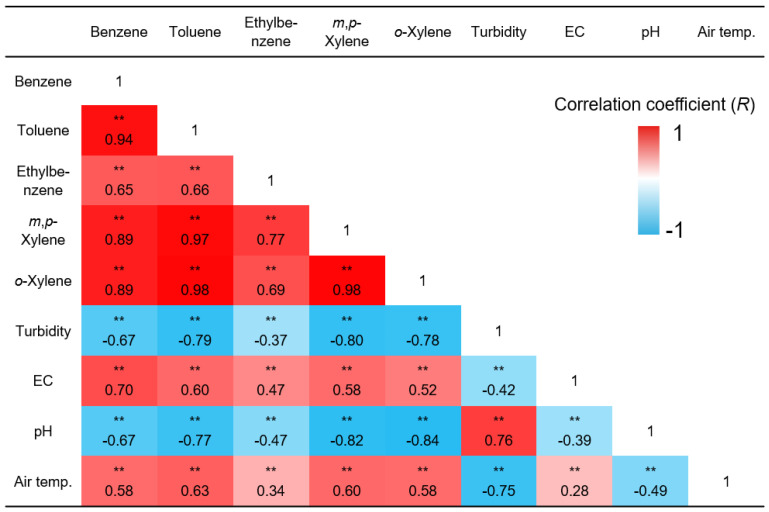
Results of correlation analysis among BTEX concentrations, turbidity, electrical conductivity (EC), and pH of groundwater, and air temperature (*n* = 115). ‘**’ indicates a relationship exhibiting a strong correlation (2-tails) for *p* < 0.01.

To better understand the factors affecting BTEX concentrations in groundwater, Pearson’s correlation analysis was performed. The detailed results of the correlation analysis are shown in Figure 8. Strong correlations (R > 0.65, *p* < 0.01) among BTEX concentrations were found. It indicates that the variation patterns of the concentrations of BTEX are consistent throughout the monitoring period, although factors affecting the concentrations of BTEX may vary (e.g., groundwater level, temperature, water quality, external source). It is noteworthy that a strong negative correlation between BTEX concentrations and turbidity of groundwater was observed. Natural organic matter contained in turbidity-producing substances can adsorb/absorb BTEX, which can decrease the concentrations of BTEX in groundwater [23]. The EC, pH of groundwater, and air temperature can also significantly change BTEX concentrations, but whether it is due to the effect of a single factor or a complex phenomenon, the mechanism is still unknown. Overall, the on-site monitoring system in this study enables statistical analysis of various environmental factors and contaminant concentrations, and by using this information, enables more rational and scientific site management strategy establishment.

#### 3.2.2. Unsaturated Zone

**Figure 9:**
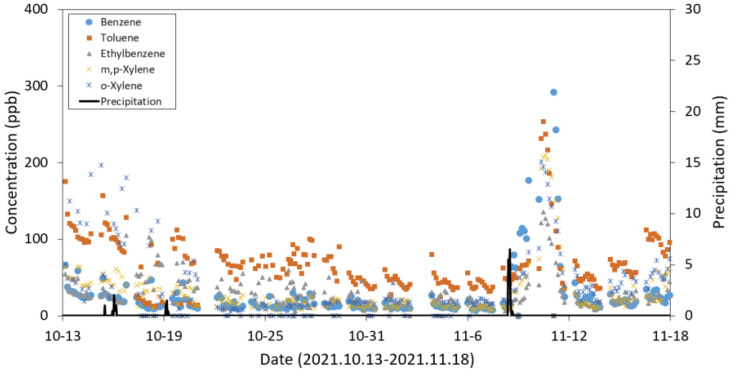
Continuous field monitoring data in the unsaturated zone (monitoring period: 13 October–18 November 2021).

The standard curve was analyzed every 2–3 days during the test period, and the monitoring systems maintained reliable conditions for BTEX analysis constantly (R^2^ > 0.98, Figure S2). The BTEX concentrations in the unsaturated zone are shown in Figure 9.

A trend of daily fluctuations of the BTEX concentrations in the unsaturated zone was seldom observed except for a few cases in toluene in which the concentration gradually decreased until noon and rebounded during the afternoon (Figure S3). The rainfall event that occurred on 8 November 2021 in the area resulted in the surge of the BTEX concentrations for a couple of days after the rainfall (Figure 9). As the groundwater table increased due to the rainfall, the BTEX, as light non-aqueous phase liquids in the groundwater, appears to be transported closer to the sampling spot located 2 m below the ground surface. The prolonged contamination might cause the LNAPL layer on top of the groundwater, which moved along with the groundwater table [24]. This causes the increased BTEX flux from the groundwater to the atmosphere due to the enhanced dispersion [25], resulting in the surge of the BTEX concentration in the unsaturated zone. In other words, shortening the BTEX migration pathway from the groundwater can lead to greater vapor intrusion [26]. This result indicates that continuous monitoring would be able to show the weather effect, especially rainfall, and the resultant increase in the VOCs of interest underground. The decision-making process with regard to the vapor intrusion originating from the groundwater surface should be based on the data acquired under various weather conditions, especially with various rainfall intensities.

## 4. Conclusions

The continuous on-site VOC monitoring system, which was developed by combining TD and GC/FID, was successfully applied to oil-contaminated sites. TD was employed as a pretreatment step to concentrate VOC samples to facilitate separate analysis of the BTEX components using GC/FID. The on-site monitoring system assisted in acquiring time-series VOC concentration data in the saturated and unsaturated zones. Additionally, insights that could not be confirmed with the existing “sample collection-transport-laboratory analysis” procedures could be confirmed through the newly developed systems. In particular, it turned out that the environmental effects such as daily temperature fluctuations and the heavy rainfall event are associated with the subsurface BTEX variations, which can eventually affect the vapor intrusion risk. Thus, such a continuous on-site monitoring system is essential for more reliable risk assessment and establishment of remediation actions for VOC contamination in soil, air, and water environments.

Although the continuous on-site monitoring system was separately developed and operated for the saturated and the unsaturated zones in this study, the feasibility of the development and operation of the TD-GC merged system is worthwhile to investigate further. Moreover, with the simultaneous recording of other factors, such as weather conditions and groundwater quality parameters, this monitoring system would be more powerful in providing a time-series dataset, which would be key to determining the fate and transport of VOCs in the subsurface.

## Supplementary Materials

The followings are available online at https://www.mdpi.com/article/10.3390/ijerph19063400/s1. Table S1: BTEX concentrations measured in the unsaturated zones before the monitoring system was installed; Table S2: (a) Precision and detection limits, and (b) accuracy of the developed monitoring system for BTEX in groundwater. Relative standard deviation (RSD), limit of detection (LOD), and limit of quantification (LOQ) were calculated using the repeated analysis results of the 2 μg/L BTEX working standard; Table S3: Precision and accuracy of the developed monitoring system for standard 100 ppb BTEX gas for the flowrate of (a) 50 mL/min, (b) 100 mL/min, and (c) 150 mL/min; Figure S1: Overview of the sampling to the monitoring system installed at the site; Figure S2: BTEX standard curves measured in the monitoring system at the field (measured on (a) 19 October 2021, (b) 31 October 2021, and (c) 9 November 2021); Figure S3: Daily fluctuations of air temperature and toluene concentration in the unsaturated zone (monitored on 1 November 2021 and 4 November 2021).
