# Supplementary material for: Continuous VOCs Monitoring in Saturated and Unsaturated Zones Using Thermal Desorber and Gas Chromatography: System Development and Field Application
Source: Int J Environ Res Public Health. 2022 Mar 14;19(6):3400. doi: 10.3390/ijerph19063400 (PMC8950982; doi:10.3390/ijerph19063400)
Supplement: Supplementary file 1 [file ijerph-19-03400-s001.zip › ijerph-1620596-supplementary.pdf]

Supplementary Materials

Continuous VOCs Monitoring in Saturated and Unsaturated Zones Using Thermal Desorber and Gas Chromatography: System Development and Field Application

JINSUNG AN[1], DONGJUN BAEK[1], JISEOK HONG[2], EUNSOO CHOI[2], IJUNG KIM[2,*]

[1] Department of Biological and Environmental Engineering, Semyung University, 65 Semyung-ro, Jechoen-si, Chungcheongbuk-do 27136, Republic of Korea.

[2] Department of Civil and Environmental Engineering, Hongik University, 94 Wausan-ro, Mapo-gu, Seoul 04066, Republic of Korea

*Corresponding Author: Email: ijung.kim@hongik.ac.kr

Number of Pages: 9

Number of Tables: 3

Number of Figures: 3

Table S1: BTEX concentrations measured in the unsaturated zones before the monitoring system was installed.

	TVOC ($\mu g/m^3$)	Benzene ($\mu g/m^3$)	Toluene ($\mu g/m^3$)	Ethylbenzene ($\mu g/m^3$)	Xylene ($\mu g/m^3$)
1	42926.5	229.3	145.6	344.3	130.6
2	635.5	23.2	40.0	76.2	81.3
3	4592.9	55.3	19.0	697.3	279.3
4	28236.9	89.2	21.5	270.7	317.1

Note: Data were obtained by the sampling, transport, and analysis method – (1) sampling using the hand-held pump (MP-Σ30) and the adsorbent tube (Tenax-TA tube), and (2) subsequent TD-GC analysis in the laboratory.

Table S2: (a) Precision and detection limits, and (b) accuracy of the developed monitoring system for BTEX in groundwater. Relative standard deviation (RSD), limit of detection (LOD), and limit of quantification (LOQ) were calculated using the repeated analysis results of the 2 μg/L BTEX working standard.

(a)

Compound	RSD (%)	LOD (ppb)	LOQ (ppb)
Benzene	0.970	0.056	0.177
Toluene	0.970	0.055	0.176
Ethylbenzene	1.11	0.058	0.184
m,p-Xylene	1.41	0.075	0.238
o-Xylene	1.31	0.073	0.232

(b)

Compound	VOC concentrations in standard solution					
	10 ppb		20 ppb		40 ppb	
	Average (ppb)	Accuracy (%)	Average (ppb)	Accuracy (%)	Average (ppb)	Accuracy (%)
Benzene	10.04	100.4	19.94	99.7	40.02	100.1
Toluene	10.10	101.0	19.84	99.2	40.05	100.1
Ethylbenzene	10.11	101.1	19.84	99.2	40.05	100.1
m,p-Xylene	10.12	101.2	19.82	99.1	40.06	100.1
o-Xylene	10.19	101.9	19.72	98.6	40.09	100.2

Table S3: Precision and accuracy of the developed monitoring system for standard 100 ppb BTEX gas for the flowrate of (a) 50 mL/min, (b) 100 mL/min, and (c) 150 mL/min.

(a)

**Compound**	**Adsorption time**							
	**5 min**		**10 min**		**15 min**		**20 min**	
	**Precision (%)**	**Accuracy (%)**	**Precision (%)**	**Accuracy (%)**	**Precision (%)**	**Accuracy (%)**	**Precision (%)**	**Accuracy (%)**
Benzene	5.20	108.67	4.78	102.74	2.34	101.60	1.36	103.28
Toluene	2.87	119.41	1.21	103.12	7.36	110.75	6.71	116.12
Ethylbenzene	7.17	90.94	1.44	111.84	3.93	107.82	4.00	113.16
m,p-Xylene	9.91	88.06	1.21	112.18	4.27	108.59	4.20	115.21
o-Xylene	9.31	73.06	1.43	101.47	6.34	111.30	5.70	117.92

(b)

**Compound**	**Adsorption time**							
	**5 min**		**10 min**		**15 min**		**20 min**	
	**Precision (%)**	**Accuracy (%)**	**Precision (%)**	**Accuracy (%)**	**Precision (%)**	**Accuracy (%)**	**Precision (%)**	**Accuracy (%)**
Benzene	6.17	107.80	2.27	104.85	3.15	100.55	2.45	98.82
Toluene	1.73	117.81	3.67	115.57	4.81	102.27	2.83	100.14
Ethylbenzene	4.50	92.60	2.98	93.87	5.58	93.93	2.74	98.51
m,p-Xylene	4.66	98.58	3.38	95.96	5.90	95.44	2.67	100.05
o-Xylene	2.79	99.60	6.38	92.98	6.48	92.59	2.81	95.61

(c)

Compound	Adsorption time							
	5 min		10 min		15 min		20 min	
	**Precision (%)**	**Accuracy (%)**	**Precision (%)**	**Accuracy (%)**	**Precision (%)**	**Accuracy (%)**	**Precision (%)**	**Accuracy (%)**
Benzene	2.79	104.50	3.18	103.07	1.73	100.40	1.56	101.88
Toluene	2.44	102.47	3.27	104.48	1.87	100.17	1.08	106.43
Ethylbenzene	2.26	117.95	1.65	104.15	1.32	104.30	1.02	105.95
m,*p*-Xylene	3.44	119.81	2.05	104.69	1.82	105.20	1.16	107.52
o-Xylene	5.39	120.20	3.03	101.32	5.27	103.49	2.68	109.16

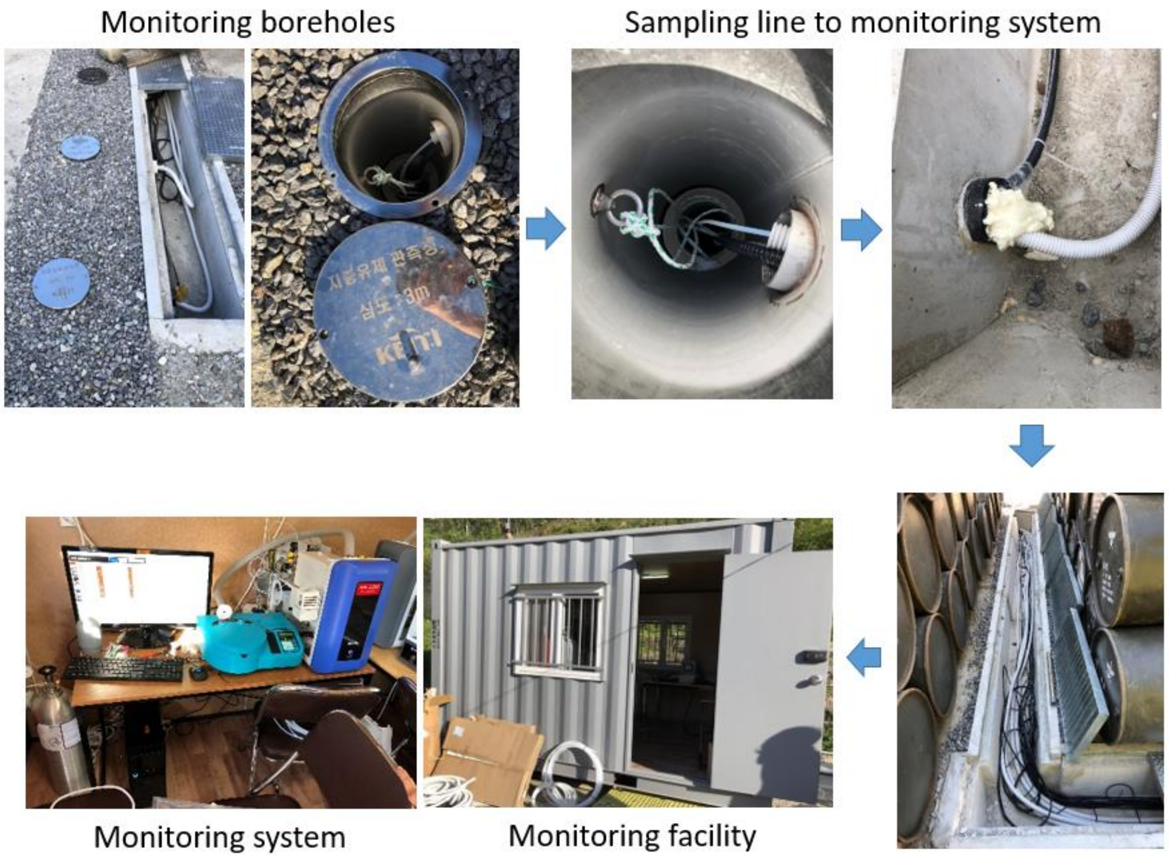

Figure S1: Overview of the sampling to the monitoring system installed at the site.

(a)

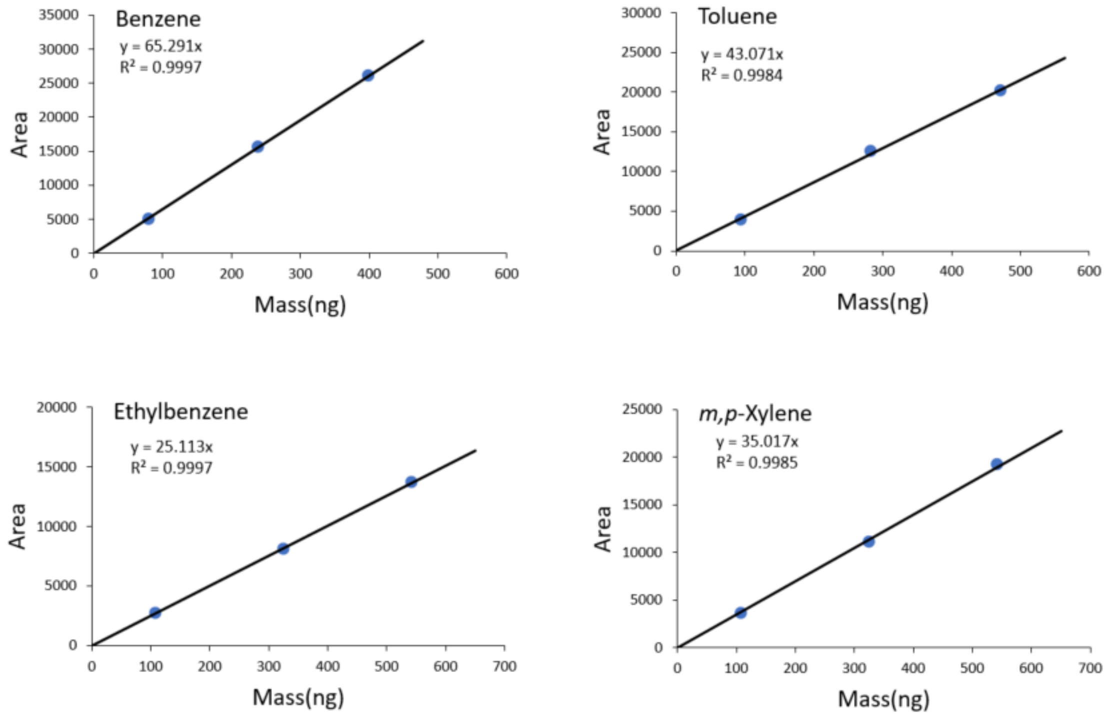
Benzene
y = 65.291x
R² = 0.9997
Area
Mass(ng)
Toluene
y = 43.071x
R² = 0.9984
Area
Mass(ng)
Ethylbenzene
y = 25.113x
R² = 0.9997
Area
Mass(ng)
m,p-Xylene
y = 35.017x
R² = 0.9985
Area
Mass(ng)

(b)

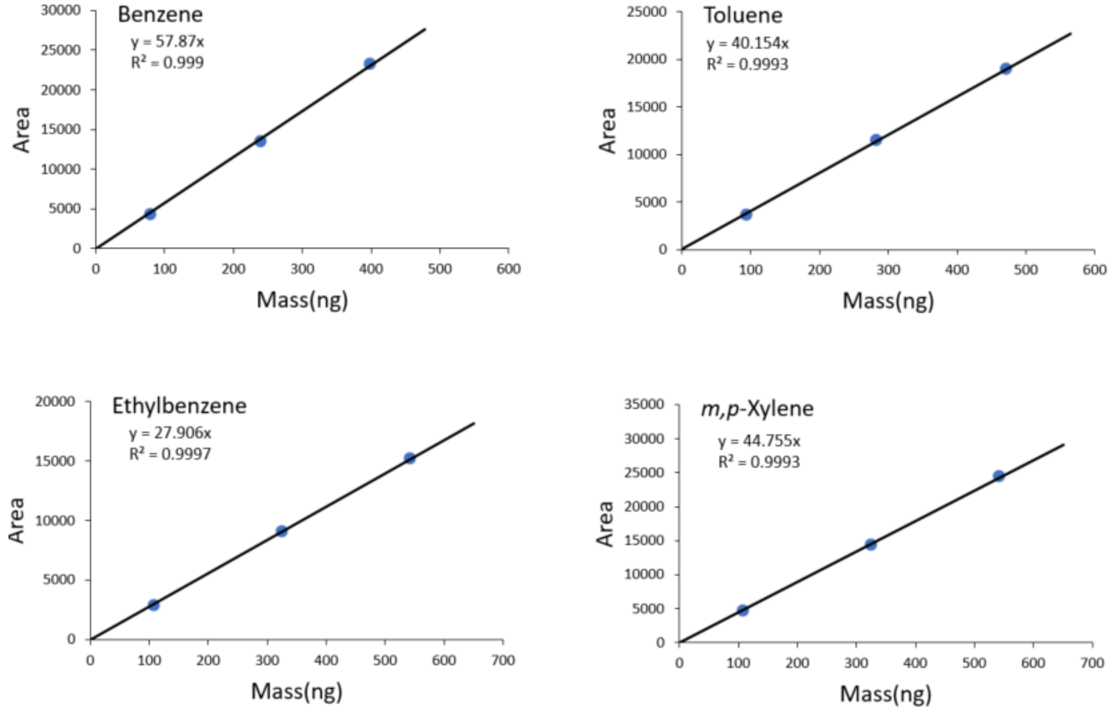
Benzene
y = 57.87x
R² = 0.999
Area
Mass(ng)
Toluene
y = 40.154x
R² = 0.9993
Area
Mass(ng)
Ethylbenzene
y = 27.906x
R² = 0.9997
Area
Mass(ng)
m,p-Xylene
y = 44.755x
R² = 0.9993
Area
Mass(ng)

(c)

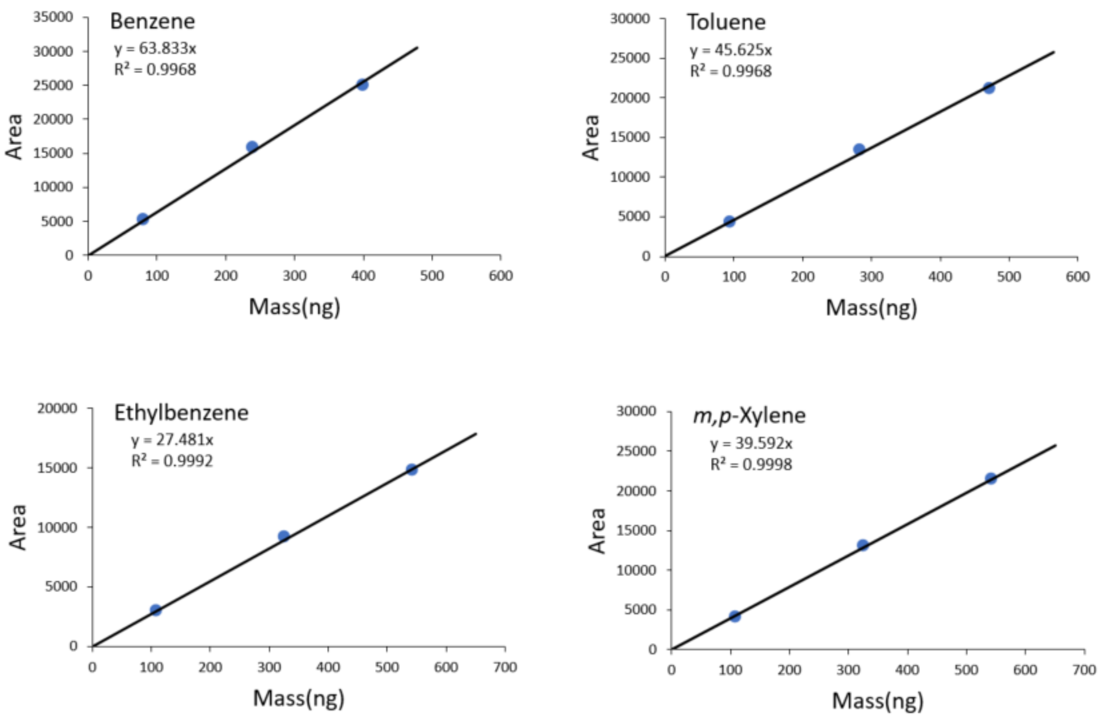

Figure S2: BTEX standard curves measured in the monitoring system at the field (measured on (a) October 19, 2021, (b) October 31, 2021, and (c) November 9, 2021).

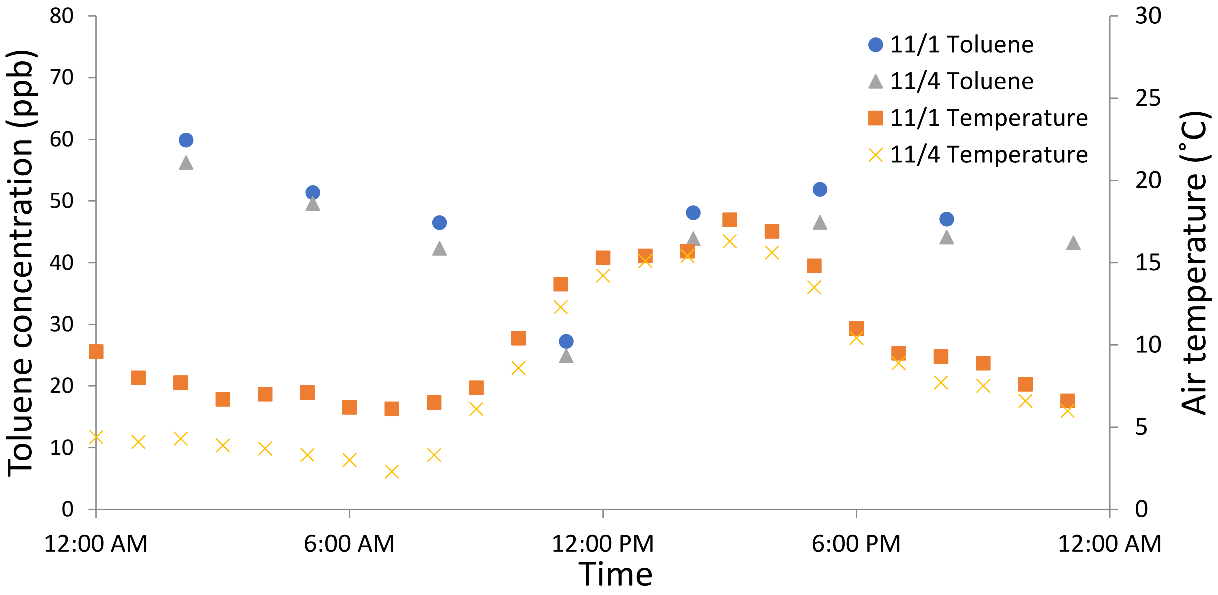

Figure S3: Daily fluctuations of air temperature and toluene concentration in the unsaturated zone (monitored on November 1, 2021 and November 4, 2021).
